# The Committee Reports on Meat Inspection and Actinomycosis

**Published:** 1892-03

**Authors:** Olaf Schwarzkopff


### THE COMMITTEE REPORTS ON MEAT INSPECTION AND ACTINOMYCOSIS.

By Olaf Schwarzkopff.

(*Continued.*)

Dr. Williams, in the above arguments* in favor of the contagiousness of actinomycosis, takes as granted certain premises which will not be accepted by every one. His interpretation of the Peoria cases, where "the extension of the disease *seemed* due to the fact that badly affected animals were kept in the sheds with the healthy" (page 541, this Journal), can reasonably be disputed. Dr. Williams concludes that here inoculation took place from the purulent discharge of the actinomycotic abscesses containing actinomyces. Others, however, will believe that the cattle were exposed to a common cause, and that here the actinomyces adhered, either to the grain or the "very coarse, hard wild hay" he mentions as being fed, which explanation is far more probable on botanical grounds. Prof. Boström some time ago announced the result of careful examinations made of actinomycotic tumors of cattle (32 cases), and man (12 cases)! In the great majority of these cases he has been successful in tracing the cause of the tumor to kernels of grain, fragments of straw, hay, etc., which he found in the starting point of the tumor, and which were profusely infested with actinomyces. These observations are confirmed by Saltmann, Bertha, Lunor, Schartau and Fischer, all of whom publish similar observations. In the face of these facts, only the theory of intertransmission from animal to animal by cohabitation becomes highly improbable.

Quite as rash is Dr. Williams in his conclusions as to the value of experimental inoculation of actinomycosis, which he calls the "crucial test" of contagiousness. The practice of experimental inoculation originated in the desire to continue the study of pathological changes of mycotic diseases in the laboratory, and small animals were found to be particularly adapted to this purpose. It is true that it has been demonstrated in this way that some contagious diseases may be artificially transplanted, bnt there are also exceptions which break down the theory that such is a sure test for contagiousness. Pleuro-pneumonia in cattle, for instance, has never been transmitted to any experimental animal, still it is justly

* February Number of Journal.

considered a contagious disease. On the other hand we may inoculate a rabbit with the cocci of pus from an abscess and produce a severe disease and perhaps death, but nobody would look upon an abscess as contagious. Perhaps this example may seem ridiculous, nevertheless, this and similar instances are facts which are not counted by some overzealous experimenters. With due regard, therefore, for the utility of artificial inoculation, it cannot, *eo ipso*, be accepted as proof of the true nature of a disease, nor does it explain the natural way in which the micro-organism enters into the body.

But suppose the inoculation theory of Dr. Williams were correct, how are we to explain the large majority of failures to transmit actinomycosis experimentally. Even in this country where little has been done in this respect up to date, the results are mostly negative. Dr. R. R. Dinwiddie, of the Arkansas Agricultural Experiment Station, in his report on actinomycosis, says : "Cultivation of the fungus on artificial media outside the body, has not been successful in my hands, nor have I succeeded in producing the disease in healthy animals by inoculation!" Dr. Bodamer (Journal, Vol. X., No. 2, 1889) reports very interesting experiments with dogs, cats, and rabbits. Although he claims to have succeeded in producing "marked lesions in six out of thirteen animals," a careful analysis of his records rather leaves one in doubt as to whether he produced genuine actinomycosis, as he only says, pus from fistula contains actinomyces. He undoubtedly saw this difficulty, and modestly says on page 120 : "But the experiments, I confess, are not altogether satisfactory, not being repeated in sufficient number. . . . Control-experiments with other than actinomyces-material were not made, because I had witnessed in the investigations of Dr. Formard very numerous experiments with *simple irritants* to terminate in precisely the same manner as my above stated experiments, etc."

My own experiments, 16 in number (3 dogs, 2 cats, 3 calves, 8 cattle), were entirely negative, except in the calf of the cow from which I used the virus for inoculation! But after making an incision into the artificially produced tumor of the calf for microscopical examination, it gradually disappeared without any treatment whatever, and nothing but a slight scar is left of it, and the animal enjoys to-day—two years after the inoculation—the best of health. From these experiments which were skillfully performed, and with the earnest desire to inform myself of the transmissibility or non-transmissibility of actinomycosis, I can frankly say that I

have formed the firm opinion that it is difficult and at most times impossible to artificially produce true actinomycosis by inoculation from animal to animal, and that I cannot thus see how the natural propagation of the disease can possibly take place by intertransmission of animals as advocated by Dr. Williams. His conclusion is the more unfortunate, as he delights in comparing actinomycosis with tuberculosis and glanders, which diseases radically differ from actinomycosis at least in two points, namely, in the readiness to cultivate the bacilli, and in the result of experimental inoculation, which is so easily attained as to be useful for controlling a diagnosis.

But as critics may demand further evidence, it may be well to relate more cases of unsuccessful inoculation, and these are given in abundance by European experimenters. In fact they are so numerous that I must refrain from enumerating them here, and can only refer to the latest contribution on actinomycosis by Prof. Boström, in Ziegler's Beiträge zur Pathologischen Anatomie, neunter Band, 1891, which is, so far, the most complete treatise on this question.

Considering the matter as a whole from what has been published about actinomycosis during the last year, it appears that all our great authorities are becoming more and more of the opinion that actinomycosis is not a malignant disease. This view was very apparent at the last meeting of the International Congress of Hygiene in London, in August, 1891, and I shall shortly quote what some of these men had to say there about actinomycosis.

*The Lancet*, London, August 22, 1891. Section III.—Relation of diseases of animals to those of man.—*Actinomycosis, Prof. Crookshank*. . . . With regard to intercommunicability of the disease between different species of animals, including man, he had failed to find any evidence in support of the theory from clinical observation. He was *not* of the opinion that the disease was contagious, in the ordinary sense of the word. *Prof. Ponfick* (Breslau), said that he agreed in the main with Prof. Crookshank's conclusions. In cattle the disease was generally transmitted by means of the fodder, especially straw. He thought that in both man and animals the source was the same, and that the disease did not spread directly from one to the other. Prof. Nocard (Alfort), said that the observations of Prof. Crookshank had been confirmed by all observers. The geographical distribution of the disease was very irregular. Bavaria, Scotland, Italy, and some of the Northern States of America had been much affected. In France it was very rare, and generally remained in an isolated form. He had never seen cases transmitted by contagion or infection. The disease appeared to him undoubtedly to spread by means of certain food-stuffs. The wholesale destruction of the flesh of the affected animals appeared to him to be quite unnecessary. *M. Dayen* (Rheims), read a paper on three cases of actinomycosis in man, which he describes at length. He maintains that not only by the courses of the disease, but also by the different appearances of the fungus, one should be able to distinguish between the bovine and the human forms. As regards the etiology, these three cases all came from the environs of Rheims ; they lived in the country, and one of them had acquired a habit of chewing grains of oats and barley. *Sir Henry Simpson* remarked that in his district the disease was not endemic, and when imported, though it did not cease with the animals imported, yet it did not spread like a contagious disease. As to whether the meat from these carcases was fit for human consumption, he was content to permit its sale, provided the locally diseased parts were cut away. *Dr. George Fleming* commented on the grave doubts which existed of the direct communicability of this disease to the human species ; if, therefore, an animal were in good condition and showed no fever, then its flesh was good to eat, but the actually diseased parts should not go into market. *Prof. Crookshank* in reply said, that in comparing the disease with tuberculosis, it should be remembered that was not, by any means, so virulent as tuberculosis.

These short extracts are certaily valuable as they show us the moderation of opinion of some of our best authorities on actinomycosis.

After having exhausted his attempts to show all the bad features of actinomycosis of man and animals, Dr. Williams becomes quite comical and produces the following tirade: (Journal, Vol. xii, No. 10, Page 541).

"A long list of leading scientists of the day might be quoted, who believe in the contagiousness of actinomycosis, such as Bollinger, Ponfick, Johne, Friedberger, Frohner, Rosenbach, Bizozzero, Lindquist, Hleller, Peroncito, Ochsner, Crookshank, Fleming, Liautard, Law and others, almost without number. In fact, it seems in so far as your chairman has been able to learn, that contributors alike to standard and current veterinary literature of a recent date, all are agreed that the affection is contagious. We understand that Prof. Schwarzkopff and other dissenters have given voice to their theoroies and deductions through the columns of the agricultural or live-stock press, and avoided placing their views in form or place where it would come within proper range of scientific criticism. We trust that now for once Prof. Schwarzkopf and his colleagues will present their non-congious theory of actinomycosis before this convention, from a scientific standpoint, and permit their arguments to be weighed upon a strictly scientific as well as practical basis. We desire that Prof. Schwarzkopf should fully explain his statement at our last meeting that he predicated his belief of the non-contagiousness of actinomycosis on his theoretical studies, and his practical work in the great abbatoirs of Berin. What great veterinarians of Berlin taught our fellow-committeeman that actinomycosis was non-contagious, while such Germans as Johne, Ponfick, Rosenbach, Friedberger, and such veterinarians of the great Berlin thierarztliche Hochschule as Frohner unitedly and without fear or apology denominate the disease as infectious? What facts has he learned in the slaughter houses of Berlin that demonstrate the non-transmissibility of the disease?"

To this I have the following to say: Bollinger and Friedberger are my teachers, and neither of them has ever said that actinomycosis is contagious, which may be stated to their honor. Ponfick, Johne, Frohner, Peroncito, have said that actinomycosis is infectious, by which they mean that the micro-organism has its natural origin outside the animal body on vegetable life, but if introduced into the body, accidentally produces disease. Crookshank and Fleming have similar opinion. Rosenbach, Bizozzero, Lindquist, Heller, are unknown to me. Liautard and Law have pronounced the disease contagious, for which I have no explanation to offer, except that Prof. Liautard holds a different opinion to-day.

I have not "avoided placing my views in form or place where it would come within proper range of scientific criticism," as I have had no fears to cross swords with Dr. Williams on scientific questions. The fact is that our American veterinary journals paid no attention to actinomycosis, but this was done by the better agricultural press. The *Breeders' Gazette* in commenting upon the pamphlet No. 1 of the Illinois Live-Stock Commissioners in their issue of March 5, 1890, called forth several articles of Prof. James Law, who strongly advocated the contagiousness of actinomycosis, and it was not until he rejected—in a somewhat left-hand way—the statements of my former chief Dr. Hertwig, of Berlin, that I participated in the discussion in the *Breeders' Gazette*, defending Hertwig's position. This was public writing and could be followed by any veterinarian that keeps acquainted with our agricultural press.

My theoretical studies and practical work in the great slaughter-houses of Berlin have naturally shaped my opinion of actinomycosis. Dr. Williams seems not to be aware that the Berlin abbatoir has a bacteriological laboratory which, superintended by Director Hertwig, its energetic chief-veterinarian, is in charge of Dr. Dunker, a well-known microscopist, and who discovered the actinomyces of the muscles in the hog. From 1883-1885, while being employed there, numerous fruitless attempts were made to cultivate the actinomyces in ordinary ways, together with inoculation-experiments which likewise failed to throw any light on the peculiarities of the fungus. We naturally gained the impression from these experiments, and by the information gathered from rural districts affected with the disease, that it could not be pronounced contagious.

The practical conclusion from what I have tried to explain in the foregoing pages certainly warrants the necessity of changing the modus operandi of some of our sanitary inspectors in the stock-yards of our country. I am strongly of the opinion that it is the duty of the sanitary veterinarian to *rather preserve meat than to waste or destroy it.* Sentimentalism, such as the argument of the abhorrence of the American for meat from animals not strictly sound, is out of the way, as it throws a veil over the naked facts of science. It may be pardonable if uttered by a layman, but it is inexcusable in a veterinarian that has intelligently studied the theory and practice of sanitary science. Of course, we should condemn an animal that is suffering from general actinomycosis, which, however, is very rare. But it is unjust—alike to producer and consumer—to reject meat of good quality from animals because they have showed so visible an affection as lumpy-jaw, whereas, we may daily consume meat of animals suffering from a more or less dreadful disease, such as we find in the slaughter-house only, but which is naturally overlooked under our present system of inspection of the living animal only.

I sincerely hope that the extreme views and teachings on actinomycosis by some of our American college professors, which are largely the cause of the erroneous views accepted by many of our superficially educated veterinarians, may become more moderate; for there is nothing to gain from obstinately declaring actinomycosis to be a contagious and dangerous disease, but much to be lost in spreading broadcast such alarming professional opinions which are at once injurious to our cattle industry and to the scientific standing of the veterinary profession of this country.

